# Development of a Reliable ic-ELISA with a Robust Antimatrix Interference Capability Based on QuEChERS Technology for the Rapid Detection of Zearalenone in Edible and Medical Coix Seeds and Subsequent Risk Assessments

**DOI:** 10.3390/foods11192983

**Published:** 2022-09-24

**Authors:** Kaiyi Guan, Rentang Huang, Hongmei Liu, Yuxin Huang, Ali Chen, Xiangsheng Zhao, Shumei Wang, Lei Zhang

**Affiliations:** 1 Key Laboratory of Digital Quality Evaluation of Chinese Materia Medica of State Administration of TCM and Engineering & Technology Research Center for Chinese Materia Medica Quality of Guangdong Province, Guangdong Pharmaceutical University, Guangzhou 510006, China; 2 Academy of National Food and Strategic Reserves Administration, Beijing 100037, China; 3 School of Chemistry and Chemical Engineering, Guangdong Pharmaceutical University, Guangzhou 510006, China; 4 Key Laboratory of Resources Conservation and Development of Southern Medicine of Hainan Province & Hainan Branch of the Institute of Medicinal Plant Development, Chinese Academy of Medical Sciences and Peking Union Medical College, Haikou 570311, China

**Keywords:** mycotoxin, extraction method, matrix effect, deterministic approach, dietary exposure

## Abstract

Indirect competitive enzyme-linked immunosorbent assay (ic-ELISA) is an ideal immunoassay method for large-scale screenings to detect mycotoxin contaminants. However, the matrix effect of complicated samples has always been challenging when performing immunoassays, as it leads to false-positive or negative results. In this study, convenient QuEChERS technology combined with optimizing the dilution solvent was ingeniously used to eliminate interference from the sample matrix to greatly improve the detection accuracy, and reliable ic-ELISAs for the two official tolerance levels of 60 and 500 μg/kg were developed to screen zearalenone (ZEN) in edible and medical coix seeds without any further correction. Then, the 122 batches of coix seeds were determined, and the positive rate was up to 97.54%. The contaminated distribution was further analyzed, and risk assessment was subsequently performed for its edible and medical purposes. The findings indicated that consumption of coix seeds with higher ZEN contamination levels may cause adverse health effects for both medical and edible consumption in the adult population; even under the condition of average contamination level, ZEN from coix seeds was the more prominent contributor to the total risk compared to other sources when used as food; thus, effective prevention and control should be an essential topic in the future.

## 1. Introduction

Mycotoxins are secondary metabolites produced by fungal species such as Aspergillus, Alternaria, Claviceps, Penicillium and Fusarium. Of the known mycotoxins, zearalenone (ZEN), which is primarily generated by Fusarium molds, is regarded as a serious problem worldwide, as it can lead to reproductive and fertility disorders by competing with 17β-estradiol for estrogen receptor binding [1].

Coix seed, known as Job’s tears, belongs to the grass family Poaceae and is a minor grain that is extensively cultivated in East and Southeast Asia (China, Japan, Thailand, India, Korea and Burma) [2]. Coix seeds are rich in amino acids, protein, lipids, fiber, calcium, iron, and vitamin B1 and have been recommended as a nourishing food for routine health care [3,4]. Coix seeds are also an important medicinal herb for excreting dampness and invigorating the spleen, and coix seed extract has been used as a raw material for the “Kanglaite Injection” anticancer drug [5]. However, numerous studies have indicated that coix seeds are prone to be contaminated by ZEN and have a higher incidence of contamination than that of other grains [6,7,8]. To date, two official tolerance levels of 60 and 500 μg/kg for ZEN in coix seed have been established by United States Pharmacopoeia’s Herbal Medicines Compendium and the Chinese Pharmacopoeia, 2020 edition, respectively. In addition, the maximum residue limit (MRL) of ZEN was established by the European Commission to be no more than 100 μg/kg in unprocessed grains [9] and 60 μg/kg in grains and their products in China [10]. Thus, more attention has been given to a simple and reliable analytical method to monitor ZEN contamination in coix seeds.

Indirect competitive enzyme-linked immunosorbent assay (ic-ELISA) is an ideal immunoassay method for large-scale screening to detect mycotoxin contaminants [11,12,13]. However, the matrix effect that occurs with complicated samples has always been a challenge, in which ic-ELISA results in false-positive or negative results [14]. To date, the commonly used method for reducing the matrix effect is diluting the sample extract [14,15], but the sensitivity of the detection method decreases as the dilution factor increases. Another general method is using a matrix-matched calibration curve for correcting the interference from the matrix effect, and this has been the most reported method in previous immunoassay studies [16,17]. However, the matrix-matched calibration curve always involves the accompanying blank sample extraction, which adds to the complexity of the detection. Coix seeds are rich in esters, polysaccharides, flavonoids, alkaloids, starch and other contents that are prone to induce matrix effects [18]. A magnetic bead microprobe-based immunoassay was developed by Liu [17] for ZEN detection, and it was also observed that the matrix-matched competitive calibration equation had to be applied for complex coix seed matrices. Therefore, a simple and effective sample pretreatment method for eliminating or reducing the matrix effect of coix seeds in ZEN detection by ic-ELISA is urgently needed.

QuEChERS (Quick, Easy, Cheap, Effective, Rugged, and Safe) is an extraction and clean-up technique that was initially developed to determine pesticide residue by gas chromatography with mass spectrometry (GC–MS/MS) [19] and then has gradually been applied to detect mycotoxins by liquid chromatography–tandem mass spectrometry (LC–MS/MS) in recent years [20,21]. It is well known that the advantages of QuEChERS include significantly eliminated matrix effects, simplicity, inexpensiveness, low reagent consumption, environmental friendliness, and shorter time of analysis compared with that of other clean-up methods [18,22]. The convenient QuEChERS-based procedure does not strikingly increase the analysis time; therefore, this procedure is worth trying with rapid screening methods such as ELISA. Unfortunately, limited studies have been conducted in recent years.

In this study, a reliable ic-ELISA method for ZEN in coix seeds was developed. A combination of an extremely simple QuEChERS pretreatment strategy and the optimization of the dilution solvent can effectively eliminate the matrix effect in real sample analyses. The proposed method was devised for both official tolerance levels of ZEN in coix seeds by adjusting the dilution parameters, the method was applied to investigate the contamination of ZEN in 122 batch coix seeds, and the results were further validated by LC–MS/MS. Moreover, health risk assessment was performed to help clarify the present situation of ZEN contamination and the potential health risks of coix seeds.

## 2. Materials and Methods

### 2.1. Reagents and Materials

The mycotoxin standards ZEN, α-zearalenol (α-ZEL) and β-zearalenol (β-ZEL) were purchased from Pribolab (Singapore). ZEN monoclonal antibody and ZEN-BSA were purchased from Shenzhen Anti Biological Technology Co., Ltd. (Shenzhen, China). Goat anti-mouse IgG polyclonal antibody-HRP (IgG-HRP) was purchased from Nanning Blue Light Biotechnology Inc. (Nanning, China). Bovine serum albumin (BSA) was obtained from Phygene Biotechnology Co., Ltd. (Fuzhou, China). The 3,3′,5,5′-tetramethylbenzidine (TMB) substrate reagent set was purchased from Bosf. (Hefei, China). Methanol and acetonitrile were provided by Macklin (Shanghai, China). Sodium acetate (CH_3_COONa), anhydrous magnesium sulfate (MgSO_4_) and other chemical reagents were of analytical grade and were from Guangzhou Chemical Reagent Factory (Guangzhou, China).

### 2.2. Sample Preparation

One hundred and twenty-two batches of coix seeds (including 22 batches of powdered coix seed) were collected from supermarkets and drugstores in China. The samples (except powdered coix seed) were ground through a high-speed disintegrator (Yongkang Sufeng Industry and Trade Company Limited, Zhejiang, China) and passed through a 24-mesh sieve. The coix seed sample powder (1.0 g) was added to 5 mL of 80% acetonitrile. The mixture was vortexed for 3 min and then vigorously stirred with 1.0 g anhydrous magnesium sulfate and 0.25 g sodium acetate for 1 min [23]. Then, the mixture was centrifuged at 10,000 rpm for 5 min. After centrifugation, the supernatant was diluted 20-fold in water with 10% methanol or 200-fold in phosphate-buffered saline (PBS) with 10% methanol and was centrifuged again. The supernatant was analyzed by ic-ELISA.

### 2.3. ic-ELISA Determination

The 96-well microplates were coated (100 μL per well) with ZEN-BSA in sodium carbonate coating buffer at 4 °C overnight. Then, the plates were washed three times with PBST (PBS containing 0.05% Tween 20) and blocked at 37 °C for 2 h. Each plate was washed two times with PBST. ZEN standard solution or diluted sample extract (50 μL per well) was added simultaneously along with anti-ZEN antibody (50 μL per well) at 37 °C for 1 h. Then, the plates were washed three times with PBST. IgG diluted with PBS (100 mM phosphate, pH 7.4) was added to the plates. After incubating for 1 h at 37 °C, the plates were washed four times, and 100 μL per well of the TMB/H_2_O_2_ substrate solution (5 mL of 0.7 mg mL^−1^ TMB, 10 μL of 30% H_2_O_2_, 5 mL of substrate buffer (0.05 M citrate-phosphate buffer, pH 5.0)) was added for 10 min. Finally, the reaction was terminated by hydrochloric acid, and the absorbance was determined at 450 nm with a spark multimode microplate reader.

### 2.4. LC–MS/MS Confirmation

The method was based on that described by Wu [8] with modifications. The sample was separated by an LC (Agilent 1290) system. A Waters CORTECSTM UPLC C18 column (100 mm × 2.1 mm × 1.6 μm) was maintained at 35 °C. The mobile phase comprised water with 0.1% formic acid and 1 mM ammonium acetate (A) and methanol (B). The gradient condition was as follows: 90% A at 0–2 min, 10% A at 2–8 min, 90% A at 8.1 min, and then returned to the initial phase at 8.1–11 min. The injection volume was 2 μL, and the flow rate was 0.3 mL/min. The spectral detection was operated with an electrospray ion (ESI) source in negative ionization mode with a capillary voltage of 3.5 kV, and multiple reaction monitoring (MRM) was utilized. The gas temperature was set at 350 °C.

### 2.5. Risk Assessment

The risk assessment is discussed separately in this work because coix seed is used for both edible and medical purposes.

The estimated daily exposure (EDE) was determined using mycotoxin contents in coix seeds and food consumption data for the adult population (Equation (1) [24]).

(1) $$\mathrm{EDE}=\frac{\left(C_{m}\times K\right)}{\mathrm{bw}}$$

where C_m_ refers to the concentration of ZEN (μg/kg), K represents coix seed consumption and is expressed in kilograms per day, and bw is the average weight of adults (70 kg).

The risk related to ZEN exposure from daily consumption of coix seeds was evaluated by calculating the ratio (% TDI) between the estimated daily exposure (EDE) and the tolerable daily intake (TDI) (Equation (2)).

(2) $$\%\mathrm{TDI}=\frac{\mathrm{EDE}}{\mathrm{TDI}}\times 100$$

As food, the daily consumption of coix seeds was set to 0.0252 kg according to Zhu’s survey of coix seed consumption in Shanghai, China [25]. As a medicinal herb, the daily consumption of coix seeds was set to 0.03 kg, according to the Chinese Pharmacopoeia 2020 edition. It is generally recognized that Chinese medicines are taken for 90 days a year, and the frequency of consumption in a year is 0.247 [26]. Hence, Equation (1) needs to be multiplied by the frequency of consumption (f). The TDI value of 0.25 μg/kg bw/day for ZEN, established by the European Food Safety Authority (EFSA) in 2011, was used [27].

The %TDI value < 100% is assumed to be within the acceptable range, equal to or exceeding %TDI may concern potential non-carcinogenic effects. Note, however, that the risk will increase as %TDI increases [28,29].

## 3. Results and Discussion

Considering the two MRLs (60 μg/kg or 500 μg/kg) of ZEN in coix seeds, we tried to adjust the different sample dilution processes to make the concentration of MRLs around the inhibitory concentration 50% (IC_50_) of the calibration curve, which ensures that the measurements are more accurate when the contamination levels of ZEN are near the MRLs in real samples. Therefore, the optimization and subsequent validation of the developed ic-ELISA were carried out separately for these two MRLs.

### 3.1. Sample Preparation

#### 3.1.1. Extraction Based on QuEChERS Technology

The solvent is critical for facilitating the extraction of ZEN. In general, methanol and acetonitrile are the most frequent extraction solvents for ZEN [30]. Previous research indicated that acetonitrile as the extraction solution could reduce fat-soluble impurities and improve the extraction efficiency [31]. Considering that the coix seed is rich in starch, some water should be mixed with the organic solvent to improve permeability, but as the proportion of water increases, the composition of the matrix becomes more complicated. Thus, in this work, 80% acetonitrile was used as the extraction solvent.

**Figure 1:**
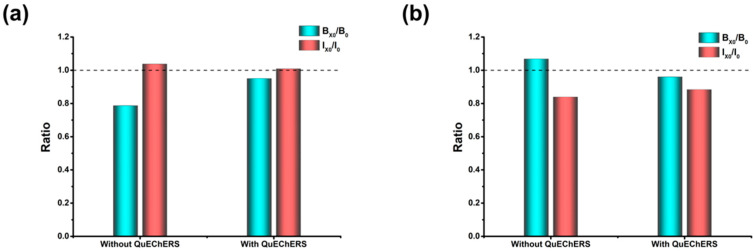
The comparison of extraction with or without QuEChERS procedure. (**a**) MRL 60 μg/kg; (**b**) MRL 500 μg/kg. The I_0_ and I_X0_ were the inhibition values of solvent and matrix at 0.5 ng/mL, respectively; the B_0_ and B_X0_ were the values of blank solvent and blank matrix, respectively. The ratio was the value of B_X0_/ B_0_ or I_X0_/I_0_.

In ELISA analysis, one-step extraction combined with subsequent dilution was the commonly used method for sample pretreatment. However, there was some interference in both the absorbance and sensitivity of the detection with direct dilution after extraction in our study. Therefore, a simple QuEChERS technology was used to improve the matrix effect. In detail, after extraction with 80% acetonitrile for 3 min, a mixture of anhydrous magnesium sulfate (MgSO_4_) and sodium acetate (CH_3_COONa) was added, and the sample was vortexed in situ for 1 min. After centrifugation, ZEN could be transferred into the organic phase, while some polar components of the matrix remained in the aqueous layer. It is worth mentioning that this QuEChERS procedure could better reduce the matrix effect both in the absorbance and inhibition rate compared to the direct dilution procedure, especially for the dilution of MRL 60 μg/kg (Figure 1). Compared to the conventional methods for minimizing matrix interference in mycotoxin extraction, such as liquid-liquid extraction [32], immunoaffinity column cleanup [33] and matrix-matched calibration [31], the proposed QuEChERS extraction is obviously more convenient, cheap and time-saving. Hence, the subsequent optimization was performed based on the QuEChERS procedure.

#### 3.1.2. Dilution

**Figure 2:**
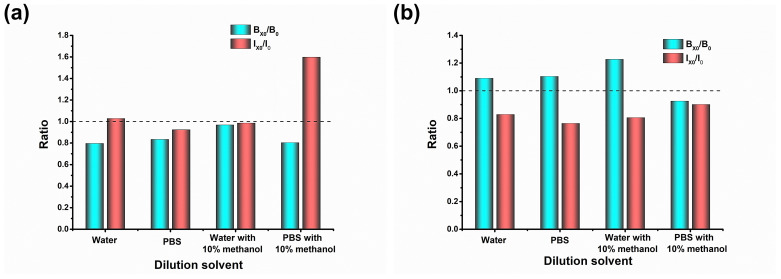
The screening of dilution solvent. (**a**) MRL 60 μg/kg; (**b**) MRL 500 μg/kg. The I_0_ and I_X0_ were the inhibition values of solvent and matrix at 0.5 ng/mL, respectively; the B_0_ and B_X0_ were the values of blank solvent and blank matrix, respectively.

The dilution ratio is commonly considered and optimized to reduce the matrix effect [14,34]. Obviously, the greater the dilution ratio is, the lower the sensitivity of the method, and the application of the detection method will be limited in situations without an ultrahigh sensitivity. In this work, we focused more on screening the dilution solvent, which has often been overlooked in previous studies. The 4 dilution solvents (water, PBS, water with 10% methanol and PBS with 10% methanol) were compared. It is interesting that, as shown in Figure 2, the inhibition rate was more affected for an MRL of 60 μg/kg, as well as the strong influence on the absorbance of an MRL of 500 μg/kg. Finally, water with 10% methanol at 60 μg/kg and PBS with 10% methanol at 500 μg/kg were selected.

### 3.2. Validation of ic-ELISA

#### 3.2.1. Linearity

**Figure 3:**
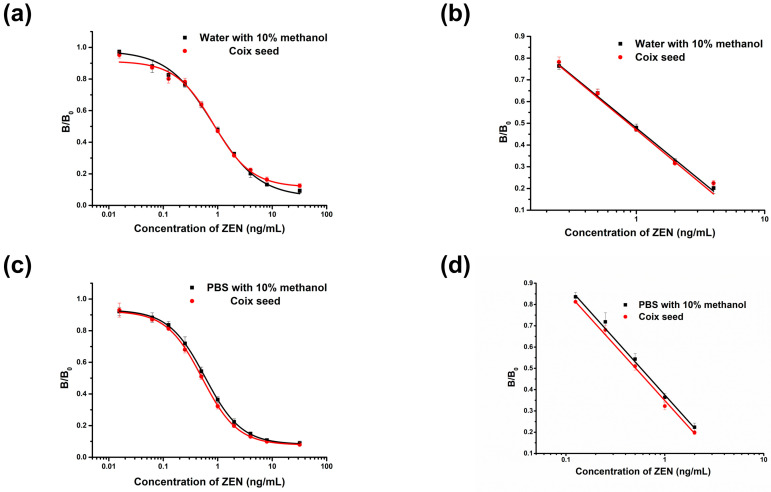
The ic-ELISA calibration curves for zearalenone (ZEN) determination in coix seed. Each data point represents the mean of three parallel experiments. (**a**,**c**) inhibition curves were generated using a four-parameter logistic fit for MRL 60 μg/kg and MRL μg/kg, respectively; (**b**,**d**) the linear parts of the curves were fitted by the least square method for MRL 60 μg/kg and MRL 500 μg/kg, respectively.

First, the matrix effect was assessed by comparing the slope of a calibration curve for standard solutions with that of matrix-matched standard solutions [35,36]. After determining the optimum conditions of ic-ELISA, inhibition curves were generated using four-parameter logistic fit (Figure 3a,c), and the linear parts of the curves were fitted by least square method to obtain linear standard curves (Figure 3b,d). The ratios of the matrix-matched standards and the solvent linear standard curve slope were 1.02 and 0.99 for the MRLs of 60 and 500 μg/kg, respectively. This result indicated that the matrix effects from the sample were within the acceptable levels of 0.85~1.15 [37]. Thus, the solvent curves could be used for detection instead of the matrix-matched curves and were quantified over the range of 16.3–400 μg/kg for an MRL of 60 μg/kg (Figure 3b) and 125–2000 μg/kg for an MRL of 500 μg/kg (Figure 3d).

#### 3.2.2. Sensitivity and Specificity

The developed method achieved different sensitivities to satisfy different detection requirements. The IC_50_ values of ZEN were 0.83 and 0.53 ng/mL for an MRL of 60 μg/kg and an MRL of 500 μg/kg, respectively (Table 1). The specificity of the ELISA between ZEN and ZEN analogs was also checked by cross-reactivity. The cross-reactivity of ZEN analogs was determined by comparing the concentrations of ZEN and its analogs, generating half-maximal inhibitions (IC_50_ for ZEN/IC_50_ for ZEN analog * 100%) (Table 1). Because of the relatively higher cross-reactivity of α-ZEL, the method may have a slightly positive bias for naturally contaminated samples.

#### 3.2.3. Limit of Detection and Limit of Quantification

The limits of detection (LOD) and quantification (LOQ), defined as the analyte concentration necessary for obtaining a 10% and 20% inhibition for ELISA [38], were 6.56 μg/kg and 16.3 μg/kg for an MRL 60 μg/kg, respectively, as well as 37.8 μg/kg and 125 μg/kg for an MRL of 500 μg/kg, respectively.

#### 3.2.4. Accuracy and Precision

Accuracy was determined at three concentration levels by spiking blank samples of coix seeds that were determined by LC–MS/MS to 30, 60, and 120 μg/kg for MRL 60 μg/kg and 250, 500, and 1000 μg/kg for MRL 500 μg/kg. The results are shown in Table 2. Precision was determined at 60 μg/kg or 500 μg/kg of spiking blank samples 6 times. The recoveries (for precision determination) averaged 114.3% and 90.01% for 60 and 500 μg/kg, respectively, and all coefficient variations were less than 9.0%. The findings demonstrated that the developed ic-ELISA can reliably monitor ZEN in coix seed samples.

### 3.3. The Contaminated Distribution and Comparison of ic-ELISA and LC–MS/MS for the Analysis of ZEN in Real Samples

**Figure 4:**
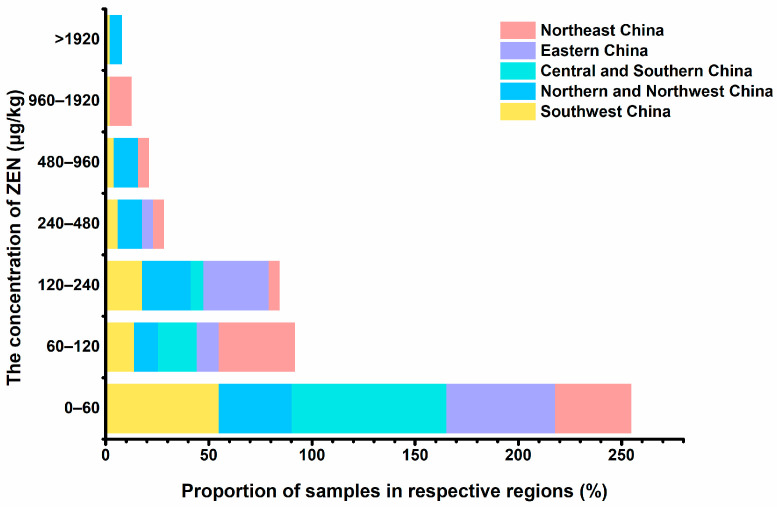
The concentration and distribution of ZEN in coix seeds. (Northeast China includes Liaoning and Heilongjiang Province; Eastern China includes Jiangsu, Zhejiang, Jiangxi, Shandong, Anhui, and Fujian Province; Central and Southern China include Henan, Hubei, Guangdong, and Guangxi Province, as well as Hong Kong and Taiwan; Northern and Northwest China include Beijing, Inner Mongolia, Hebei, Shanxi, and Shaanxi Province; Southwest China has Sichuan, Guizhou and Yunnan Province.).

**Figure 5:**
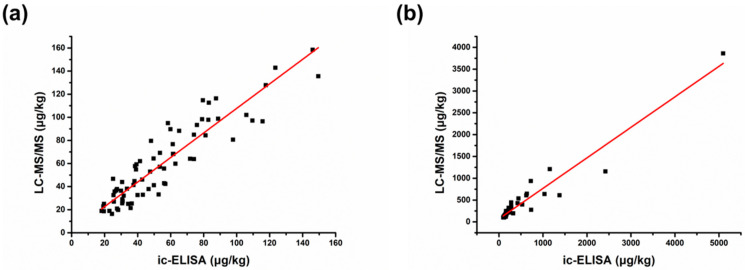
Correlation curves for ZEN in coix seed between the developed ic-ELISA and the LC-MS/MS fitted by the method of weighted least square. (**a**) MRL 60 μg/kg; (**b**) MRL 500 μg/kg.

One hundred and twenty-two batches of coix seeds were determined by the developed ic-ELISA. The contamination levels of ZEN in the samples can be seen in Table S1. Among them, the positive rate was up to 97.54% with the contamination level ranging from 17.52 to 5094.70 μg/kg, in which 51.26% exceeded the limit of 60 μg/kg and 8.40% exceeded the limit of 500 μg/kg. We also tried to draw a contamination distribution diagram (Figure 4), and it showed that more than 45% of samples in other regions had ZEN contamination levels greater than 60 μg/kg except for Central and Southern China, of which the samples in Northeast China and Northern and Northwest China had higher levels of contamination (>480 μg/kg). As previously referenced, the occurrence of ZEN in coix seeds was widespread with a higher incidence of contamination than that of other grains, and our results confirmed this (Table 3). To date, preventive measures are deficient for reducing ZEN contamination in coix seeds, which should be paid more attention to in future research. The positive results obtained were further compared with those of ultrahigh-performance LC–MS/MS, which showed a better correlation (R = 0.902/0.904) (Figure 5), suggesting that the proposed ic-ELISA was reliable and accurate.

### 3.4. Risk Assessment of ZEN in Coix Seeds

According to the higher occurrence rate and contamination levels of ZEN in coix seeds, an exposure assessment to ZEN for both medical and edible consumption of coix seeds is necessary. The exposure assessment was performed by a deterministic approach. Three scenarios were considered, including the minimum, average, and maximum concentrations of ZEN in coix seeds (Table 4).

As food, the calculated %TDI value of 733.64 for ZEN in scenario III was observed to far exceed the safety reference value of 100%. As a medicinal herb, the EDE value was about one-third of the food’s, but the calculated %TDI value of 215.72 was still much higher than 100%. The above observations indicated that consumption of coix seeds with higher ZEN contamination levels might cause adverse health effects for both edible and medicinal use. This finding was consistent with previous studies [25,28]. For scenario I, because of the %TDI obtained, 2.52 and 0.74 for use as food and medicinal herb, respectively, the risk assessment linked with the exposure to ZEN was considered lower for the studied populations. The %TDI values for ZEN in scenario II were 29.90 and 8.79 for edible and medicinal aims, respectively, indicating they were within an acceptable risk range; furthermore, the ZEN exposure derived from other foods probably co-consumed was taken into account under this condition. As shown in Table 3, the average dietary exposure of adults to ZEN in cereal and cereal products, corn and corn products, corn oil and wheat flour in China ranged between 0.003 and 0.052 μg/kg bw/day, which represents 1.2~20.92% of TDI. Thus, assuming any of the food above was consumed with coix seeds, the total ZEN exposure was calculated below the TDI. In this status, when coix seeds were used as food, ZEN from coix seeds was the more prominent contributor to the total risk compared to other sources. Based on these findings, constant monitoring of the production and storage process is required to minimize health risks related to the intake of ZEN in coix seeds.

As mentioned in Section 3.3, regional variations of ZEN contamination in coix seeds were observed. Therefore, it was speculated that there was also a regional difference in health risk associated with dietary exposure to ZEN. As shown in the contamination distribution diagram (Figure 4), the order of regions by the relative contamination severity from high to low was Northern and Northwest China, Northeast China, Southwest China, Eastern China, and Central and Southern China. According to the calculation equation of %TDI mentioned in Section 2.5, the order of regions by the relative contamination severity was consistent with the order by the health risk. That means ZEN from Northern and Northwest China was the more significant contributor to the total risk compared to other regions, while Central and Southern China was lower. However, the misreporting of estimated dietary exposure to ZEN from coix seeds in the different areas could not be excluded due to a lack of data on regional consumption. Further investigation is needed to confirm this finding.

## 4. Conclusions

In this work, a reliable ic-ELISA for the detection of ZEN with two different MRLs in coix seeds was developed without any correction. It is worth noting that we proposed a strategy to eliminate the matrix effect from coix seed samples that was problematic for previous immunoassay-based rapid detection. It was found that using simple QuEChERS technology as sample pretreatment could reduce the matrix effect both on absorbance and sensitivity. In addition, optimizing the dilution solvent could especially improve interference on sensitivity when the dilution ratio was relatively low, but when the dilution ratio was higher, this improvement was mainly observed for the absorbance. The 122 batches of coix seeds were determined, and a better correlation was obtained compared with that of ultrahigh-performance LC–MS/MS. The contamination distribution was further analyzed, and the results indicated that the occurrence of ZEN in coix seeds is a universal problem in various districts in China. Moreover, based on the edible and medical aims, the dietary exposure to ZEN for adults was assessed separately. The findings indicated concern about intake of the ZEN through consuming coix seeds with higher contamination levels. When used as food, the intake of ZEN through consuming coix seeds with an average concentration was within the acceptable risk range but identified as a significant contributor to the total risk compared to other sources. There was a regional difference in health risk associated with dietary exposure to ZEN from coix seeds. Still, the effect of different regions on the risk assessment is limited by a lack of data on regional consumption, which is worthy of further investigation.

## Figures and Tables

**Table 1:** IC_50_ values and Cross-reactivity of the ZEN and its analogues determined by the developed of ic-ELISA.

MRLs (μg/kg)	Compounds	IC_50_ (ng/mL)	Cross-Reactivity (%)
60	ZEN	0.83	100.0
	α-ZEL	0.73	113.7
	β-ZEL	3.15	26.3
500	ZEN	0.53	100.0
	α-ZEL	0.64	82.8
	β-ZEL	2.57	20.6

Note: 50% inhibitory concentration (IC_50_).

**Table 2:** Recoveries and coefficient variations for the accuracy in spiked coix seed (*n* = 3).

MRLs (μg/kg)	ZEN (μg/kg)		Recovery (%)	Coefficient of Variation (%)
	Spiked	Detected *		
60	30	30.87 ± 3.46	102.9	11.5
	60	65.70 ± 3.35	109.5	5.6
	120	142.03 ± 7.27	118.4	6.0
500	250	182.01 ± 10.85	72.80	4.3
	500	473.38 ± 5.28	94.68	1.0
	1000	1097.08± 63.50	109.7	6.4

* All data are expressed as the mean ± SD.

**Table 3:** Natural occurrence of ZEN and estimated daily exposure of adults to ZEN in common food and coix seeds samples in China.

Product	*n* *	Occurrence Rate	Mean (μg/kg)	Range (μg/kg)	EDE ** (μg/kg bw/Day)	Reference
corn	280	37.5%	121.1	4.12–1712.1	0.0238	[39]
corn oil	31	87.1%	54.4	n.d.–220.0	0.027	[40]
corn and corn products corn oil	392 63	38.27% 79.37%	12.3 149	5.20–218 14.3–516	0.004 (urban)/0.009 (rural) 0.003 (urban)/0.003 (rural)	[41]
cereal and cereal products	355	8.73%	8.19	n.d.–369	0.052	[42]
wheat flour corn products	292 347	53.42% 87.61%	5.05 40.87	0.30–55.01 0.30–942.60	0.01 0.03	[43]
coix seed	77	98.7%	242.4	1.1–1562.3	-	[8]
coix seed	147	69.39%	327.7	<1.0–9361	0.0216	[25]
coix seed	26	84.62%	-	<0.5–4075	-	[28]
coix seed	9	100%	92.1	18.7–211.4	-	[6]
coix seed	122	97.54%	207.65	17.52–5094.70	0.0748 (as food)/0.0220 (as medicinal herb)	this work

*: number of analyzed samples. **: estimated daily exposure (EDE) value (μg/kg bw/day) of mean contamination level.

**Table 4:** The estimated daily exposure to ZEN (μg/kg bw/day) and %TDI in three exposure scenarios.

ZEN Daily Exposure	Scenario I		Scenario II		Scenario III	
	P1	P2	P1	P2	P1	P2
EDE	0.0063	0.0019	0.0748	0.0220	1.8341	0.5393
%TDI	2.52	0.74	29.90	8.79	733.64	215.72

Scenario I: the lowest concentration of ZEN in the analyzed samples. Scenario II: the average concentration of ZEN in the analyzed samples. Scenario III: the highest concentration of ZEN in the analyzed samples. P1: consumption as food. P2: consumption as medicinal herb.

## Data Availability

Data are contained within the article.

## Supplementary Materials

The following supporting information can be downloaded at: https://www.mdpi.com/article/10.3390/foods11192983/s1, Table S1: ZEN contamination levels in 122 batches of coix seeds.
