# Supplementary material for: Development of a Reliable ic-ELISA with a Robust Antimatrix Interference Capability Based on QuEChERS Technology for the Rapid Detection of Zearalenone in Edible and Medical Coix Seeds and Subsequent Risk Assessments
Source: Foods. 2022 Sep 24;11(19):2983. doi: 10.3390/foods11192983 (PMC9562022; doi:10.3390/foods11192983)
Supplement: Supplementary file 1 [file foods-11-02983-s001.zip › foods-1913550-supplementary.pdf]

Development of a Reliable ic-ELISA with a Robust Antimatrix Interference Capability Based on QuEChERS Technology for the Rapid Detection of Zearalenone in Edible and Medical Coix Seeds and Subsequent Risk Assessments

Supplementary table

Suppl. Table S1. ZEN contamination levels in 122 batches of coix seeds.

Sample	Region	Province/City	ZEN contamination (μg/kg)	
No.			Ic-ELISA	UHPLC-MS/MS
1	Southwest	Guizhou	133.02	117.29
2	China	Guizhou	41.44	62.00
3		Guizhou	19.94	n.d.
4		Guizhou	150.38	147.21
5		Guizhou	220.82	321.97
6		Guizhou	22.93	18.91
7		Guizhou	29.36	n.d.
8		Guizhou	19.64	25.02
9		Guizhou	61.68	68.20
10		Guizhou	52.70	33.13
11		Guizhou	33.56	38.16
12		Guizhou	116.53	98.53
13		Guizhou	59.90	89.67
14		Guizhou	158.93	120.84
15		Guizhou	1156.09	1209.27
16		Guizhou	23.40	57.11
17		Guizhou	43.14	32.90
18		Guizhou	20.05	11.57
19		Guizhou	19.53	18.60
20		Guizhou	53.51	69.19
21		Guizhou	38.42	57.74
22		Guizhou	62.87	59.83
23		Guizhou	75.94	93.32
24		Guizhou	158.87	160.72
25		Guizhou	88.82	98.81
26		Guizhou	58.36	94.91
27		Guizhou	279.94	391.64
28		Guizhou	81.13	84.33
29		Guizhou	<LOQ	10.81

30		Guizhou	42.77	46.09
31		Guizhou	622.18	607.70
32		Guizhou	225.20	245.72
33		Guizhou	56.00	55.57
34		Guizhou	97.32	101.30
35		Guizhou	74.10	84.91
36		Guizhou	56.10	42.89
37		Guizhou	170.88	169.72
38		Guizhou	725.87	936.34
39		Guizhou	161.58	178.78
40		Guizhou	34.28	25.02
41		Guizhou	279.03	446.36
42		Guizhou	26.99	36.64
43		Guizhou	<LOQ	15.12
44		Guizhou	30.94	27.31
45		Guizhou	29.92	36.45
46		Guizhou	36.45	25.33
47[a]		Guizhou	5094.70	3862.34
48		Guizhou	123.52	142.95
49		Yunnan	27.50	20.89
50		Sichuan	27.45	37.87
51		Yunnan	324.71	194.70
52	Northern and Northwest China	Hebei	39.28	59.25
53		Hebei	30.65	25.57
54		Hebei	48.08	79.58
55		Hebei	203.02	239.00
56		Beijing	733.21	276.47
57		Beijing	2420.87	1156.55
58		Beijing	115.70	96.46
59		Beijing	30.73	28.99
60		Inner Mongolia	425.25	428.18
61		Beijing	105.89	102.04
62		Beijing	74.09	63.86
63		Beijing	281.35	327.25
64		Beijing	31.57	32.08
65		Inner Mongolia	134.55	158.34
66		Shanxi	159.98	248.68
67		Shaanxi	149.48	135.57
68		Hebei	634.89	644.40
69	Central and Southern China	Henan	n.d.	35.14
70[a]		Henan	26.02	35.61
71[a]		Henan	97.83	80.61
72[a]		Henan	37.49	41.40
73[a]		Henan	56.77	42.32
74[a]		Hubei	<LOQ	12.58
75[a]		Taiwan	<LOQ	13.82
76[a]		Taiwan	19.22	22.99
77[a]		Taiwan	49.84	41.21
78		Hong Kong	109.57	97.19
79		Guangxi	47.57	52.94
80		Hubei	28.50	9.13

81[a]		Guangdong	18.14	18.93
82[a]		Guangdong	n.d.	13.25
83		Guangdong	79.07	98.33
84		Guangdong	17.52	3.47
85	Eastern China	Fujian	34.67	14.32
86		Fujian	83.16	112.67
87		Jiangxi	25.61	26.97
88		Fujian	30.59	43.96
89		Fujian	49.63	64.34
90		Fujian	25.40	32.59
91		Fujian	224.51	215.73
92		Fujian	38.09	44.73
93[a]		Fujian	71.76	64.09
94[a]		Fujian	25.11	46.74
95[a]		Fujian	114.68	128.69
96[a]		Shandong	203.53	256.54
97[a]		Anhui	39.94	32.71
98[a]		Zhejiang	121.14	106.46
99[a]		Jiangsu	248.26	217.94
100[a]		Shandong	130.32	122.19
101[a]		Anhui	153.32	187.88
102[a]		Shandong	38.99	54.87
103[a]		Shandong	182.02	203.25
104	Northeast China	Liaoning	79.53	114.71
105		Liaoning	n.d.	1.11
106		Liaoning	1376.44	609.99
107		Liaoning	35.75	21.40
108		Liaoning	82.87	97.80
109		Liaoning	146.11	158.47
110		Liaoning	61.43	68.29
111		Liaoning	117.68	127.80
112		Liaoning	87.50	116.33
113		Liaoning	530.75	398.62
114		Liaoning	61.26	76.74
115		Heilongjiang	28.24	5.00
116		Heilongjiang	24.47	16.31
117		Heilongjiang	65.19	88.23
118		Heilongjiang	46.65	37.88
119		Heilongjiang	1036.95	637.09
120		Heilongjiang	28.17	19.82
121		Heilongjiang	444.75	538.08
122		Heilongjiang	30.70	5.61

n.d. is not detected; a means powdered coix seed.
